# Electronic π‐to‐π* Excitations of Rhodamine Dyes Exhibit a Time‐Dependent Kohn–Sham Theory “Cyanine Problem”

**DOI:** 10.1002/open.201700046

**Published:** 2017-05-02

**Authors:** Barry Moore, Robert L. Schrader, Karol Kowalski, Jochen Autschbach

**Affiliations:** ^1^ Department of Chemistry, University at BuffaloState University of New YorkBuffaloNY14260-3000USA; ^2^ William R. Wiley Environmental Molecular Sciences LaboratoryPacific Northwest National LaboratoryRichlandWA99352USA

**Keywords:** chromophores, computational chemistry, density functional calculations, electronic spectra, heterocycles

## Abstract

The longest‐wavelength π‐to‐π* electronic excitations of rhodamine‐like dyes (RDs) with different group 16 heteroatoms (O, S, Se, Te) have been investigated. Time‐dependent Kohn–Sham theory (TDKST) calculations were compared with coupled‐cluster (CC) and equations‐of‐motion (EOM) CC results for π‐to‐π* singlet and triplet excitations. The RDs exhibit characteristics in the TDKST calculations that are very similar to previously investigated cyanine dyes, in the sense that the singlet energies obtained with nonhybrid functionals are too high compared with the CC results at the SD(T) level. The errors became increasingly larger for functionals with increasing amounts of exact exchange. TDKST with all tested functionals led to severe underestimations of the corresponding triplet excitations and overestimations of the singlet–triplet gaps. Long‐range‐corrected range‐separated exchange and “optimal tuning” of the range separation parameter did not significantly improve the TDKST results. A detailed analysis suggests that the problem is differential electron correlation between the ground and excited states, which is not treated sufficiently by the relatively small integrals over the exchange‐correlation response kernel that enter the excitation energy expression. Numerical criteria are suggested that may help identify “cyanine‐like” problems in TDKST calculations of excitation spectra.

## 1 Introduction

**Figure 1:**
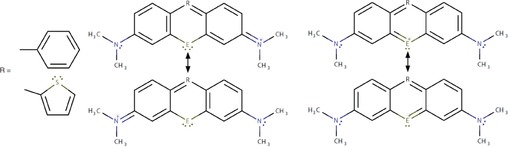
The rhodamine‐type dyes studied herein. E=O, S, Se, or Te; series **1**‐E: R=phenyl, series **2**‐E: R=thienyl. Left: Cyanine‐like and right: anthracene‐like resonance structures.

Dyes based on derivatives of the rhodamine core (Figure 1) have numerous applications in biochemistry, biotechnology, physical chemistry, and related fields.^1^, ^2^, ^3^, ^4^, ^5^, ^6^, ^7^ It is, therefore, important to be able to model the photophysical properties and, chiefly among those, the electronic spectrum of a rhodamine‐type dye (RD) by using accurate and efficient quantum‐chemical methods, for example, to predict spectral changes upon chemical substitutions or to assign measured spectra. Time‐dependent Kohn–Sham theory (TDKST) linear response is often the method of choice for the calculation of electronic spectra of RDs and other π‐conjugated organic chromophores.^8^, ^9^

Typically, RDs have rather low triplet yields due to a lack of heavy atoms that would facilitate intersystem crossing through spin‐orbit (SO) coupling. The classic rhodamine core includes oxygen as a heteroatom (E). A 2007 study reported the synthesis and characterization of RDs with E replaced by one of the heavier Group 16 elements (S, Se, or Te), in addition to the oxygen analogues,^7^ with the potential of increased triplet yields (Figure 1). In the study, a decreasing energy that corresponds to the peak of the longest‐wavelength absorption band was noted across the E=O, S, Se, Te series, that is, as the atomic number of E increased. The same trend was also obtained for the first singlet excitation energies calculated by using TDKST. Analysis of the electronic structures showed that as the atomic number of E increased, the participation of the lone pairs on E in the conjugated π system decreased. A simple model was then proposed, in which the ground state has the largest weight of the anthracene‐like resonance structures (Figure 1) when the E conjugation is strongest (i.e. for O) and the smallest weight when the E conjugation is least pronounced (i.e. for Te). The opposite trend would be expected for the weight of the cyanine‐like resonance structures. The anthracene‐like resonance structures have a shorter pathlength for π conjugation than the cyanine‐like ones and are, therefore, expected to contribute to higher excitation energies.

Cyanine dyes have been extensively studied in recent years for their noted unusual behavior in calculations of electronic spectra by using TDKST, namely, that the longest‐wavelength transitions are calculated to be significantly too high in energy.^10^, ^11^, ^12^, ^13^, ^14^, ^15^, ^16^, ^17^ Often, TDKST underestimates excitation energies, in particular when the excitation has pronounced charge‐transfer (CT) character and when nonhybrid functionals are used. This type of error is well understood and has been linked to the KS delocalization error.^18^, ^19^, ^20^ The cyanine excitations are of the π–π* type and primarily involve the highest occupied molecular orbital (HOMO) and lowest unoccupied molecular orbital (LUMO) without any indication of CT or sizable delocalization errors. Moreover, the overestimation of the excitation energies becomes worse on going from nonhybrid functionals to functionals with increasing fractions of exact exchange (eX). An early theoretical study found that complete active space self‐consistent field calculations with dynamic correlation treated by second‐order perturbation theory (CASPT2) produced good agreement with the experimental band peak positions,^21^ but Hartree–Fock plus configuration interaction singles and TDKST linear response strongly overestimated these energies. The authors noted the difficulties in finding a rationale for this failure because no substantial multireference character of the ground‐state or double‐excitation character of the excitations was found. The usual approximate KS functional and adiabatic exchange‐correlation (XC) response kernels in TDKS are known to be inadequate to treat such problem cases. Subsequent research has indicated that cyanine and related transitions for π‐chromophores without any indication of physical CT appear to afford a large amount of differential correlation between the ground state ($S_{0}$ ) and first singlet excited state ($S_{1}$ ),^11^, ^14^, ^16^, ^22^ and that commonly used approximate TDKST linear response XC kernels treat this differential correlation insufficiently. Most notably for the cyanine dyes, the energy gaps between the $S_{1}$ states and the lowest‐energy triplet excited state ($T_{1}$ ), that is, the singlet–triplet (S/T) gaps, are much too large in TDKST calculations, with the triplet excitation energies (${}^{3}\Delta E=E\left(T_{1}\right)-E\left(S_{0}\right)$ ) being underestimated. In a recent benchmark of S/T gaps,^23^ the authors observed a similar underestimation of triplet excitation energies for molecular semiconductors. According to our analysis of Ref. 14, a partial cancellation of the errors in the S/T gap and in ${}^{3}\Delta E$ then leads to the calculated singlet excitation energies (${}^{1}\Delta E=E\left(S_{1}\right)-E\left(S_{0}\right)$ ) to be the least overestimated when using nonhybrid XC functionals.

We also identified similar issues with singlet transitions in other types of organic chromophores that had previously been associated with a “CT‐like” problem.^16^ Various types of diagnostics were applied in Ref. 16, such as an assessment of how the energy differences associated with the TDKST linear response kernel compared with a Δ SCF‐type result (SCF=self‐consistent field), comparisons of full TDKST with the Tamm–Dancoff approximation (TDA) for triplet excitations, and various measures devised to identify CT. A key aspect of this previous research was that improved benchmark data for the vertical electronic excitation energies were generated by using coupled‐cluster (CC) theory, which included singles, doubles, and perturbative triples (CCSD(T)) for the $S_{0}$ and $T_{1}$ total energies and equation‐of‐motion CCSD(T) for ${}^{1}\Delta E$ . Particularly for cyanines and related problem cases, the perturbative triples contributions offered large corrections.

The aforementioned RD study in Ref. 7 also noted a severe overestimation of the first vertical singlet excitation energies calculated by using TDKST and hybrid functionals, as compared with the experimental longest‐wavelength absorption peak positions. Along the series E=O, S, Se, Te, a good linear correlation between the calculated ${}^{1}\Delta E$ value and the experimental band peak energies was obtained, but with slopes of 1.25 instead of 1 for the computational model that gave the best correlation, by using a hybrid with 50 % eX. Functionals with lower fractions of eX produced less of an overestimation on average, but larger slopes and worse correlation coefficients for the linear fits. Knowing that RDs have cyanine‐like resonance structures, we decided to learn whether TDKST calculations indeed suffer from a similar problem. Other candidates in which cyanine‐like problems have been diagnosed are BODIPY dyes^17^ and cationic dyes that are structurally very similar to the RDs considered herein.^24^ As in Ref. 16, comparisons with experimental band‐peak positions, which may differ from the exact vertical electronic excitation energies for a variety of reasons, are avoided. Instead, we generated CCSD(T) and EOM‐CCSD(T) benchmark data for truncated models of the dyes to assess the quality of the singlet and triplet excitation energies and the S/T gap calculated by using TDKST with different functionals. Our main conclusion is stated in the article title, namely, that the cyanine and rhodamine‐type family of dyes do indeed exhibit very similar behavior as far as the approximations in the TDKST linear response excitation spectra calculations are concerned.

## Computational Details

Relaxed rotation profiles (RRPs) for the rotation of the R groups were obtained for the two series of dyes in Figure 1 by using Gaussian 09.^25^ For consistency in the E series, heteroatoms O, S, Se, and Te were described by large‐core^26^, ^27^ Stuttgart relativistic effective core potentials (ECPs) with matching optimized valence basis sets. The triplet‐ζ valence polarized basis^28^ (TZVP) from the Environmental Molecular Sciences Laboratory basis set exchange^29^ was used for all other atoms. Kohn–Sham theory (KST) optimizations were carried out with the B3LYP^30^ functional with ultrafine grids and very tight convergence criteria defined in Gaussian. The RRP‐optimized structures were subsequently truncated to facilitate comparisons with the computationally rather demanding CCSD(T) and completely renormalized (CR) EOM‐CCSD(T) calculations,^31^ by replacing the ‐CH_3_ groups of the ‐N(CH_3_)_2_ moieties and the R groups with hydrogen atoms, followed by optimization at the aforementioned level in *C*_2*v*_ symmetry.

System‐specific functional tuning and calculations with TDKST and CC were performed by using a 2016 developer version of NWChem.^32^, ^33^ The tuning determined an optimized range‐separation parameter in the exchange functional of a long‐range‐corrected (LC) version of the Perdew–Burke–Ernzerhof PBE XC functional^34^ nonempirically, to minimize the KST delocalization error and potentially improve the computed excitation energies. Tuning data can be found in Tables S5 and S6 of the Supporting Information. Extensive literature is available on this subject; details can be found in Refs. 35, 36, 37, 38. TDKST calculations by using the PBE, B3LYP, LC‐PBE, and LC‐PBE* XC functionals (* denotes the tuned parametrization) and eX‐only (i.e. Hartree–Fock (HF)) were performed by using the usual “random‐phase approximation” (RPA) version of TDKST and the Tamm–Dancoff approximation (TDA).^39^ Two‐level calculations were enabled by using the freeze keyword in NWChem, which only allowed the π and π* frontier orbitals to describe the excitation.

Various CT criteria were evaluated numerically, as described in Ref. 16, by using an in‐house library to manipulate volume data in the Gaussian cube file format (available upon request). The frontier MOs and electron‐density cube files were generated by using NWChem on a grid of 151×151×151 points that extended 2 Å beyond the smallest and largest x , y , and z coordinates of the input structures. Various additional integrals of the XC response kernel for the frontier MOs were generated by using a developer version of the Amsterdam density functional (ADF) code^40^ with the Slater‐type TZP basis set and PBE orbitals, by using an add‐on code previously developed by one of the authors.^14^, ^16^, ^41^ These integrals are based on the Vosko–Wilk–Nusair (VWN) local density approximation functional.^42^ Test calculations performed in a previous study,^14^ which compared results for two‐level models by using these integrals with two‐level NWChem PBE calculations, showed that the VWN‐based XC response kernel integrals are quite similar to those generated by using the generalized gradient approximation (GGA) PBE functional. Because we are only using these integrals for semi‐quantitative comparisons‐based two‐level models, differences in the correlation functionals are not deemed to be critical. Two‐electron repulsion integrals (ERIs) for selected pairs of orbitals were calculated by using the “twoel” code in the ADF suite, originally developed by Becke and Dickson.

Supporting Information contains relaxed rotation profiles for benzene and thiophene dyes, including optimized dihedral angles; comparisons of the singlet and triplet excitation energies of RRPs and symmetrized geometries; optimally tuned $\gamma^{*}$ for full and truncated dyes; numerical analysis for full dyes, similar to Table 4; two‐level singlet and triplet excitations described in Section 2. Additional plots are available for most of the data discussed in Section 2.

## 2 Results and Discussion

Table 1 provides the experimental absorption band peak energies for the two series of dyes, along with TDKST singlet excitation energies (${}^{1}\Delta E$ ; the Δ CCSD(T) column is explained below). The reader is reminded that * indicates the molecule‐specific tuned parametrization of the LC‐PBE hybrid. All calculations reproduced the heteroatom trends and the differences between the **1**‐E and **2**‐E series of dyes, with increasingly overestimated trends for functionals with large amounts of eX. Compared with the experimental band peak energies, the TDKST calculations moderately to severely overestimated the singlet excitation energies with increasing errors on going from the nonhybrid functional to pure HF. Tuning of the LC functional offered only relatively minor improvements. The functional trend is similar to that for cyanines, except that previous computations for cyanines found no improvements by tuning the range‐separation parameter in LC functionals.^14^

**Table 1:** Experimental and calculated singlet excitation data [eV] for the dyes series shown in Figure 1.^[a]^

	Experiment	Δ CCSD(T)^[b]^	PBE	LC‐PBE*	LC‐PBE	HF
**1**‐E						
O	2.25	0.50	2.52	2.80	2.98	3.52
S	2.17	0.49	2.44	2.70	2.87	3.38
Se	2.13	0.49	2.41	2.67	2.84	3.34
Te	2.08	0.50	2.37	2.62	2.79	3.27
**2**‐E						
O	2.18	0.57	2.44	2.72	2.90	3.43
S	2.10	0.56	2.36	2.65	2.79	3.29
Se	2.06	0.56	2.33	2.60	2.76	3.24
Te	2.02	0.56	2.30	2.55	2.71	3.18

[a] Experimental band peak positions (in methanol) from Ref. 7. [b] Δ CCSD(T) was computed as CCSD(T)(truncated dye)−experiment, with the CCSD(T) singlet energy from Table 2.

The experimental positions of the longest‐wavelength absorption peaks may or may not correspond very closely to the calculated vertical electronic excitation energies. Therefore, rather than basing our analysis on a TDKST‐versus‐experiment comparison, a theory‐versus‐theory comparison has been made for the vertical excitation energies that correspond to the longest‐wavelength intense transition. Theoretical reference values were generated from CCSD(T) calculations of the energies of the lowest singlet and spin‐triplet states, and CR‐EOM‐CCSD(T) for the singlet excitation energies, along with calculations without the perturbative triples. For the full dyes, with the TZVP basis, the CCSD(T) level of theory turned out to be too demanding of computational resources and, therefore, comparisons were made for truncated models of the dyes, as explained in the Computational Details Section. This truncation eliminated the distinction between the **1**‐E and **2**‐E series.

The intense transition in question is of $B_{1}$ symmetry in the $C_{2v}$ point group of the truncated dyes. In most cases, this transition is assigned to the HOMO–LUMO π–π* pair of orbitals. For some functionals with no or small percentages of HF and the heavier heteroatoms, an E lone‐pair orbital may be higher in energy than the π orbital involved in the transition. In this case, the intense π–π* transition is HOMO−1–LUMO, whereas an excitation with slightly lower energy and low intensity is the *n*–π* transition. We focus on the intense π–π* transition; the calculated data are collected in Table 2. The truncation of the dyes introduced a practically constant blueshift of approximately 0.33 eV for the **1**‐E series and the PBE functional, and an almost constant blueshift between 0.34 and 0.37 eV for the tuned LC‐PBE* functional parametrizations (Table S3 in the Supporting Information). The variations in these blueshifts are smaller than the changes in the experimental band peak energies on going from E=O to Te and, therefore, the model compounds are suitable for the TDKST versus CC benchmark. Moreover, the CC singlet $B_{1}$ excitation energies are at a nearly constant difference from the experimental band peak positions (blueshifted by 0.49–0.50 eV from **1**‐E and 0.56–0.57 eV from **2**‐E; see the Δ CCSD(T) column in Table 1). Therefore, the CCSD(T) level calculations model the E=O to Te trend accurately for the chromophores.

**Table 2:** Excitation data [eV] for truncated dyes at various levels of theory.

	PBE	LC‐PBE*	LC‐PBE	HF	CCSD	CCSD(T)
^**1**^ **Δ*E***						
O	2.86	3.14	3.22	3.69	3.09	2.75
S	2.77	3.05	3.13	3.56	3.00	2.66
Se	2.74	3.02	3.10	3.52	2.96	2.62
Te	2.70	2.99	3.06	3.47	2.92	2.58
^**3**^ **Δ*E***						
O	1.82	1.88	1.85	1.72	2.28	2.23
S	1.81	1.87	1.82	1.59	2.26	2.21
Se	1.81	1.86	1.81	1.55	2.25	2.19
Te	1.79	1.83	1.78	1.50	2.22	2.17
**S/T gap**						
O	1.04	1.26	1.37	1.97	0.81	0.52
S	0.96	1.18	1.31	1.97	0.74	0.45
Se	0.93	1.16	1.29	1.97	0.53	0.43
Te	0.91	1.16	1.28	1.97	0.83	0.41

The dye truncations led to a smaller blueshift in the calculated TDKST data than the difference between experimental peak positions and the CC calculations. If we assume that the truncation effect in the CC calculations would be close to that in TDKST, this difference indicates a constant residual error in the CC calculations or a constant difference between the hypothetical exact theoretical vertical excitation energies versus the experimental band peak positions. Solvent effects may also play a role because the solvent in the experiments, methanol, is quite polar. However, a continuum model applied in the calculations of Ref. 7 lowered the excitation energies only very slightly.

Table 2 provides the singlet and triplet excitation energies for the truncated dyes with the two CC levels and different functionals. The relevant singlet/triplet energy differences are also provided (S/T gaps). For convenience, the HF label refers to the eX‐only functional in the TDKST calculations, which is the same as time‐dependent Hartree–Fock (TDHF). First, it is worth noting that the ${}^{3}\Delta E$ values are slightly improved by the (T) corrections in the CC data, but not dramatically so. In contrast, the ${}^{1}\Delta E$ values are more than 0.3 eV lower with CR‐EOM‐CCSD(T) than without the triples corrections. These changes are not as large as for some of the chromophores that we studied previously, but also far from negligible. This means that for the singlet excitations of RDs, CCSD is not suitable to assess the accuracy of density functional methods for the RDs. From benchmark calculations of the excitation energies of small‐to‐medium‐sized organic molecules,^43^ it seems likely that the more efficient approximate CC2 and CC3 models may give better results than CCSD and CCSD(T), respectively. (Indeed, a test calculation for the truncated E=O dye gave a singlet/triplet excitation energy of 2.92/2.32 eV (TZVP basis).) In Ref. 43, as in our study, improvements in triplet excitation energies with higher‐order CC were modest. Conversely, many of the singlet excitations had double‐excitation character according to CASPT2 calculations, and were not well described by using CC2. The CC calculations performed for the present study did not indicate significant double‐excitation character or problems with the single reference. It is worth stressing that CCSD(T) and CR‐EOM‐CCSD(T) are potentially not accurate enough over iterative triples and beyond. However, we assume that the CC data with inclusion of perturbative triples are sufficiently accurate to assess the errors in the other calculations.

Looking at Table 2, TDKST systematically overestimates the ${}^{1}\Delta E$ value; the nonhybrid functional PBE is the closest to the CC benchmark data and HF is the furthest away. The LC hybrids are between these two extremes. The LC functional tuning offers little improvement. These trends are the same as for the calculations of the full dyes versus experimental band peak positions. For the ${}^{3}\Delta E$ values, Table 2 demonstrates a severe underestimation with PBE and even more so with HF. The tuned LC functional offers slight improvements, but, as for the singlet excitations, not to such a degree that they appear to solve the underlying problem. Therefore, the delocalization error and the associated and well‐understood CT problem of TDKST are not the main sources of error.

Table 3 shows several criteria introduced previously to gauge the extent of CT in excitations, evaluated for the truncated dyes. The spatial overlap (O ) between the π and π* orbital is sufficiently large that it does not flag a CT problem (which would be unexpected in the first place). However, the density change criterion $\frac{1}{2}\left\langle \left|\Delta \rho \right|\right\rangle$ for the two‐orbital model shows that a substantial amount of electron density is shifted between spatially disjointed regions. In conjunction with a sufficiently large spatial overlap O , we previously noted that a sizable $\frac{1}{2}\left\langle \left|\Delta \rho \right|\right\rangle$ criterion may be an indicator for large differential correlation between the two states associated with the two orbitals, similar to the cyanines. The difference between the last two columns of Table 3 indicates that there is significant relaxation of Δρ taking place when additional pairs of orbitals are allowed to participate in the excitations, even though the TDKST transition density analysis assigns the transitions as predominantly π–π*. Indeed, the two‐level singlet excitation energies are much larger, between 4.2 and 4.1 eV with PBE and between 4.1 and 4.0 eV with LC‐PBE* (Table S8). For PBE and the triplet excitations, which are practically purely π–π*, the two‐level model and full calculations agree very well.

**Table 3:** CT criteria for the truncated dye singlet excitations.^[a]^

		O	$\frac{1}{2}\left\langle \left|\Delta \rho \right|\right\rangle$ ^[b]^	$\frac{1}{2}\left\langle \left|\Delta \rho \right|\right\rangle$ ^[c]^
O	PBE	0.68	0.57	0.33
	LC‐PBE*	0.66	0.60	0.27
S	PBE	0.67	0.59	0.31
	LC‐PBE*	0.64	0.62	0.26
Se	PBE	0.67	0.59	0.31
	LC‐PBE*	0.64	0.62	0.26
Te	PBE	0.67	0.59	0.30
	LC‐PBE*	0.64	0.62	0.26

[a] O is the spatial overlap $\left\langle |\pi |||\pi^{*}|\right\rangle$ between the transition orbitals. $\frac{1}{2}\left\langle \left|\Delta \rho \right|\right\rangle$ is half of the integrated modulus of the density change upon excitation, equal to 1 for long‐range CT.^16^ [b] Corresponds to a two‐level model, using only the π–π* pair of orbitals to describe the transition density. In this case, $\frac{1}{2}\left\langle \left|\Delta \rho \right|\right\rangle$ is equal to $O{}^{'}$ , the spatial overlap between (π+π*)/√2 and (π−π*)/√2 suggested in Ref. 44 as a CT‐like criterion for symmetric dyes. [c] Using the full orbital basis to describe the excitation.

According to the data examined so far, the RDs exhibit trends that are very similar to those noted in our previous studies on cyanine transitions^14^, ^15^ and cases that had for some time been considered to be CT‐like.^16^ We advocate for abandoning the CT‐like or similar labels because a detailed analysis showed that the problem appears to have a lot in common with the cyanines and very little with physical CT. In the following, we use the term cyanine‐like instead. A distinction between cyanine‐like cases versus the RDs in this study (and cynanines) is that, for these previously studied cyanine‐like cases, nonhybrid functionals, such as PBE, underestimated ${}^{1}\Delta E$ values and a tuned LC functional brings it close to CCSD or CC2, which appears to be similar to the improvements obtained with LC functionals for actual CT excitations. We showed the effects from the triples corrections in the CC singlet energy calculations to be very important for these problematic systems, by lowering ${}^{1}\Delta E$ significantly. A comparison with CCSD or CC2 may, therefore, falsely indicate a systematic improvement in TDKST. For RDs and cyanines, the nonhybrid functional PBE already overestimates ${}^{1}\Delta E$ and the error becomes larger when eX is included in the functional, tuned or not.

The difference between the HF and CCSD(T) data in Table 2 shows the influence of the electron correlation directly. The lack of correlation has quite severe consequences that lead to an underestimation of ${}^{3}\Delta E$ by approximately 0.6 eV by HF. The ${}^{1}\Delta E$ values, conversely, are overestimated by HF theory by about 0.9 eV. It is illustrative to consider a linearized TDKST two‐orbital model, that is, the π=*p* occupied and the π*=*q* unoccupied orbital in our case. Details can be found in Ref. 16 and references cited therein, in particular Casida's article.^8^ For HF, one finds [Eqns. (1a)a), (1b)b)]:

(1a) $${\;}^{1}\Delta E^{\mathrm{HF}}=\Delta \epsilon -\left[pp\right|r_{12}^{-1}\left|qq\right]+2[pq|r_{12}^{-1}\left|pq\right]$$

(1b) $${\;}^{3}\Delta E^{\mathrm{HF}}=\Delta \epsilon -\left[pp\right|r_{12}^{-1}\left|qq\right]$$

Numerical data for the electron repulsion integrals (ERIs) and other quantities used in the discussion of the two‐orbital models can be found in Table 4 for the truncated dyes and Table S7 for the full dye. Note that the integral values depend on the functional used to generate the orbitals. For this study, PBE/TZP orbitals were used. The *p*–*q* orbital energy gap, Δϵ , is a leading term in the ${}^{1}\Delta E$ and ${}^{3}\Delta E$ expressions. Typically, with HF and eX‐rich hybrid functionals, Δϵ is larger than ${}^{3}\Delta E$ and a correction term toward the excitation energy is provided by a negative Coulomb ERI, $-[pp|r_{12}^{-1}|qq]$ , between the two orbitals (using Mulliken notation, but with the operator written explicitly). This behavior is shown by the data in Table 5, which compares the PBE, LC‐PBE*, and HF triplet excitation energies for the truncated dyes with Δϵ . The orbital energy gap is slightly higher than ${}^{3}\Delta E$ for the PBE functional, more than 4 eV higher with LC‐PBE*, and more than 6 eV higher with HF. Although contributions from other orbitals do play an important role in the overall energetics, the fact that the π–π* triplet transition in the RDs is underestimated so strongly by HF theory, and strongly but to a lesser degree by TDKST, shows both that the $-[pp|r_{12}^{-1}|qq]$ term is large and that it is too large and should be accompanied by a sizable correction from the dynamic correlation to represent the true ${}^{3}\Delta E$ values. Indeed, according to Table 4, the Coulomb integrals $\left[pp|r_{12}^{-1}|qq\right]$ are almost 5 eV (the Coulomb self‐repulsion integrals $\left[pp|r_{12}^{-1}|pp\right]$ and $\left[qq|r_{12}^{-1}|qq\right]$ well exceed 5 eV). For the full dyes (Table S7), the integrals are numerically slightly smaller but have otherwise similar characteristics.

**Table 4:** Integrals involved in the TDKST calculations of the π–π* excitation of the dye series and the two‐level models. ERIs and integrals over the XC response kernel for Slater‐type basis PBE/TZP *p*=π and *q*=π* orbitals. All values in [eV].

Integral	O	S	Se	Te
$\left[pp|r_{12}^{-1}|qq\right]$	4.899	4.792	4.763	4.722
$\left[pq|r_{12}^{-1}|pq\right]$	1.177	1.134	1.129	1.131
$\left[pp|r_{12}^{-1}|pp\right]$	5.395	5.344	5.329	5.307
$\left[qq|r_{12}^{-1}|qq\right]$	5.859	5.679	5.609	5.494
$\left[pq|f_{\mathrm{XC}}^{\alpha \alpha }|pq\right]$	−0.131	−0.123	−0.121	−0.120
$\left[pp|f_{\mathrm{XC}}^{\alpha \alpha }|pp\right]$	−0.306	−0.306	−0.305	−0.303
$\left[qq|f_{\mathrm{XC}}^{\alpha \alpha }|qq\right]$	−0.364	−0.346	−0.340	−0.329
$\left[pq|f_{\mathrm{XC}}^{\alpha \alpha }+f_{\mathrm{XC}}^{\alpha \beta }|pq\right]$	−0.151	−0.142	−0.140	−0.139
$\left[pp|f_{\mathrm{XC}}^{\alpha \alpha }+f_{\mathrm{XC}}^{\alpha \beta }|pp\right]$	−0.352	−0.351	−0.350	−0.349
$\left[qq|f_{\mathrm{XC}}^{\alpha \alpha }+f_{\mathrm{XC}}^{\alpha \beta }|qq\right]$	−0.422	−0.402	−0.394	−0.382
$\left[pq|f_{\mathrm{XC}}^{\alpha \alpha }-f_{\mathrm{XC}}^{\alpha \beta }|pq\right]$	−0.111	−0.104	−0.103	−0.102
$\left[pp|f_{\mathrm{XC}}^{\alpha \alpha }-f_{\mathrm{XC}}^{\alpha \beta }|pp\right]$	−0.260	−0.260	−0.259	−0.258
$\left[qq|f_{\mathrm{XC}}^{\alpha \alpha }-f_{\mathrm{XC}}^{\alpha \beta }|qq\right]$	−0.306	−0.291	−0.285	−0.276

**Table 5:** Triplet excitation energy vs. π–π* orbital energy gap for truncated dyes. All values in [eV].

	PBE		LC‐PBE*		HF	
	${}^{3}\Delta E$	Δϵ	${}^{3}\Delta E$	Δϵ	${}^{3}\Delta E$	Δϵ
O	1.82	2.01	1.88	6.13	1.72	8.21
S	1.81	1.99	1.87	6.02	1.59	8.09
Se	1.81	1.98	1.86	5.97	1.55	8.04
Te	1.79	1.96	1.83	5.99	1.50	7.97

The S/T gap is given in the two‐orbital model by $2[pq|r_{12}^{-1}|pq]$ , that is, by two times the exchange ERI between the two orbitals. In the RDs, as in the cyanines, these exchange integrals are comparatively large and slightly exceed 1 eV. Indeed, the HF data in Table 2 shows that the calculated HF S/T gaps are close to 2 eV, that is, about twice the exchange ERI, even though the excitation energies include contributions from other orbital pairs. Because the HF calculations severely overestimate the ${}^{1}\Delta E$ values and the S/T gap, this indicates that the dynamic correlation would need to provide a significant correction to $2[pq|r_{12}^{-1}|pq]$ to counteract these overestimations.

The linearized TDKST expressions that match Equations (1) for nonhybrid funtionals are [Eq. (2a)a); Eq. (2b)b)]:

(2a) $${\;}^{1}\Delta E^{\mathrm{KS}}=\Delta \epsilon +\left[pq\right|f_{\mathrm{XC}}^{\alpha \alpha }+f_{\mathrm{XC}}^{\alpha \beta }\left|pq\right]+2[pq|r_{12}^{-1}\left|pq\right]$$

(2b) $${\;}^{3}\Delta E^{\mathrm{KS}}=\Delta \epsilon +\left[pq\right|f_{\mathrm{XC}}^{\alpha \alpha }-f_{\mathrm{XC}}^{\alpha \beta }\left|pq\right]$$

In the expressions above, $f_{\mathrm{XC}}^{\sigma \sigma {}^{'}}$ are the spin (σ )‐dependent components of the KS linear response XC kernel. In ours and most other calculations, this kernel is frequency‐independent and local, that is, the integrals go over just one spatial electron coordinate. As in the linearized HF expressions, the orbital energy gap (Δϵ ) along with two times the exchange ERI gives zeroth‐order estimates for the excitation energies, whereas the XC kernel matrix elements provide the all‐important corrections to arrive at the (in principle, exact) excitation energies. Of course, contributions from other orbital pairs can also provide crucial contributions to these energies.

Δϵ values for molecules calculated with approximate KS functionals, in particular with nonhybrid functionals, tend to be smaller than the TDKST ${}^{1}\Delta E$ values. This is also the case for the RDs herein. For the triplet excitations, the Δϵ are slightly above the TDKST ${}^{3}\Delta E$ values calculated by using the nonhybrid functional PBE (Table 5). The XC‐kernel integrals for the RDs entering Equations (2) tend to be quite small overall, even for orbital pairs with comparatively large Coulomb and exchange ERIs. We previously showed this to be the case for cyanines and related problem cases. Table 4 shows that for the truncated series of dyes, the $\left[pq|f_{\mathrm{XC}}^{\alpha \alpha }+f_{\mathrm{XC}}^{\alpha \beta }|pq\right]$ values are −0.15 eV or smaller in magnitude, and the $\left[pq|f_{\mathrm{XC}}^{\alpha \alpha }-f_{\mathrm{XC}}^{\alpha \beta }|pq\right]$ counterparts are −0.11 eV or less. This means that in pure KS TDKST, the exchange‐correlation contributions to the excitation energies provided by response kernels derived from common approximate XC functions are unlikely to be large enough to describe excitations with significant differential correlation between the ground and the excited state that go along with large Coulomb and exchange ERIs between p and q (in less‐demanding cases, favorable results may benefit from error cancellation). With hybrid functionals there may be favorable error cancellation if the error trends from the eX fraction in the exchange potential and kernel balance those of the KS XC potential and kernel. With the nonhybrid functionals, the ${}^{3}\Delta E$ values do not include the large $-[pp|r_{12}^{-1}|qq]$ contribution present in the HF expressions. The small XC kernel integrals do not produce a dramatically different ${}^{3}\Delta E$ value than the estimate from Δϵ alone. In the PBE calculations, the latter are actually in better agreement with the CCSD(T) benchmark than the full TDKST excitation energies. As far as the S/T gap is concerned, the XC kernel contributions, along with relaxation effects from orbital pairs not considered in the two‐orbital model, do seem to provide corrections to the eX contribution in the desired direction toward smaller values, but not nearly enough to replicate the comparatively small 0.5 to 0.4 eV S/T gaps from the CCSD(T) benchmark.

Lastly, because the S/T gap is calculated here as the difference between the singlet and triplet energies, the question arises as to whether the S/T gaps are overestimated by TDKST because the triplet energies are simply too low. It has been suggested that a possible reason for underestimated triplet energies by using TDKST with functionals with large fractions of eX could be a triplet near‐instability of the singlet reference.^45^ Peach and Tozer^46^ noted in 2012 that excitations that involve orbitals with large spatial overlap tend to have triplet near‐instabilities, and recommended use of the TDA as a remedy, in conjunction with a Coulomb‐attenuated functional. Tuned LC functionals were shown to reduce such near‐instability problems significantly when present,^47^ which may be a reason for the slight improvements (≈0.1 eV) seen in the LC* data for the RDs compared with the nontuned LC functional. Table 6 lists the singlet and triplet energies calculated by using the TDA, and the differences between the TDA and the full results. For all functionals, the ${}^{1}\Delta E$ values are overestimated even more by the TDA, by between 0.3 and 0.2 eV across the dyes and functionals. In the ${}^{3}\Delta E$ values, an increase in TDA versus full calculations is seen for increasing fractions of eX in the functional, with essentially no difference for PBE and differences of 0.8 to 0.9 eV in the HF calculations. The large differences, and over‐compensation with respect to CCSD(T), between TDA‐HF and full HF may be an indication that ${}^{3}\Delta E$ in the full calculations is impacted by a near‐instability. However, the possibility remains that the TDA‐HF data for the triplet energies may be improved for the wrong reasons because there is no actual instability.

**Table 6:** TDA excitation energies [eV] and differences with respect to full TDKST for truncated dyes.

	PBE	LC‐PBE*	LC‐PBE	HF
^**3**^ **Δ*E*(TDA)**				
O	1.84	2.02	2.08	2.49
S	1.83	2.01	2.05	2.45
Se	1.83	2.00	2.04	2.43
Te	1.81	1.98	2.01	2.40
^**1**^ **Δ*E*(TDA)**				
O	3.17	3.35	3.44	3.94
S	3.03	3.25	3.33	3.81
Se	2.99	3.21	3.30	3.77
Te	2.93	3.17	3.25	3.71
^**3**^ **Δ*E*(TDA−full TDKST)**				
O	0.02	0.14	0.23	0.77
S	0.02	0.14	0.23	0.86
Se	0.02	0.13	0.23	0.88
Te	0.02	0.15	0.23	0.90
^**1**^ **Δ*E*(TDA−full TDKST)**				
O	0.31	0.22	0.21	0.25
S	0.26	0.20	0.20	0.25
Se	0.24	0.19	0.20	0.25
Te	0.23	0.18	0.19	0.24

## 3 Conclusion

The longest‐wavelength transitions of the RDs studied herein behave very similarly to those of cyanines and, to some degree, the cyanine‐like cases studied previously: TDKST (including the eX‐only functional, that is, TDHF) calculations severely underestimate ${}^{3}\Delta E$ . For nonhybrid functionals, this error seems to be largely driven by a small Δϵ in conjunction with small XC‐kernel corrections. For HF and eX‐rich functionals, the underestimation can be associated with missing correlation effects that would otherwise dampen the effect of a large $-[pp|r_{12}^{-1}|qq]$ Coulomb integral and related terms from other orbital pairs in the excitation energy. In both scenarios, lacking or not sufficiently large correlation corrections to $2[pq|r_{12}^{-1}|pq]$ and contributions from other orbital pairs causes an overestimation of the S/T gap to such a degree that ${}^{1}\Delta E$ ends up closest to the CCSD(T) benchmark with the nonhybrid functional PBE, and becomes increasingly overestimated when the functionals become eX‐rich.

Cyanine‐like problems appear to be associated with electronic transitions that involve orbitals that have 1) spatial overlaps that are not large, but too large to flag a CT problem, 2) comparatively large values for the $\frac{1}{2}\left\langle \left|\Delta \rho \right|\right\rangle$ criterion, particularly when a two‐orbital model is considered, and 3) magnitudes of the ERIs between the two orbitals that are comparably large as those for cyanines for chromophores of similar spatial extensions. A combination of these criteria may help to identify potential problem cases with significant differential correlation between the ground and excited state, which the approximate local XC kernel contributions in the excitation energies may not be capable of treating to a sufficient degree.

## Conflict of interest

*The authors declare no conflict of interest*.

## Supporting information

As a service to our authors and readers, this journal provides supporting information supplied by the authors. Such materials are peer reviewed and may be re‐organized for online delivery, but are not copy‐edited or typeset. Technical support issues arising from supporting information (other than missing files) should be addressed to the authors.

Supplementary
