# Supplementary material for: Electronic π‐to‐π* Excitations of Rhodamine Dyes Exhibit a Time‐Dependent Kohn–Sham Theory “Cyanine Problem”
Source: ChemistryOpen. 2017 May 2;6(3):385–92. doi: 10.1002/open.201700046 (PMC5474673; doi:10.1002/open.201700046)
Supplement: Supplementary file 1 — Supplementary [file OPEN-6-385-s001.pdf]

ChemistryOPEN

Supporting Information

© 2017 The Authors. Published by Wiley-VCH Verlag GmbH & Co. KGaA, Weinheim

Electronic π-to-π* Excitations of Rhodamine Dyes Exhibit a Time-Dependent Kohn–Sham Theory "Cyanine Problem"

Barry Moore, II,[a] Robert L. Schrader,[a] Karol Kowalski,[b] and Jochen Autschbach*[a]

open_201700046_sm_miscellaneous_information.pdf

Contents

1 Relaxed Rotation Profiles S2

2 Excitation Comparison between RRP and Symmetrized Geometries S4

3 Tuning Data S5

4 Additional Excitation Data and Plots S6

1 Relaxed Rotation Profiles

To examine the rotation angle of benzene and thiophene relaxed rotation profiles were performed by fixing a dihedral angle, shown in Figure S1. The benzene and thiophene were rotated from 90° to 150° in 15° increments. The results are given in Figure S2, where the relative energy (to the lowest energy rotation) is plotted versus the dihedral angle (ϕ). The low energy frozen dihedral structure is then reoptimized with no constraints (results in Table S1). The non-perpendicular 1-O, 2-O, and 2-S dihedral angles are consistent with Reference 1.

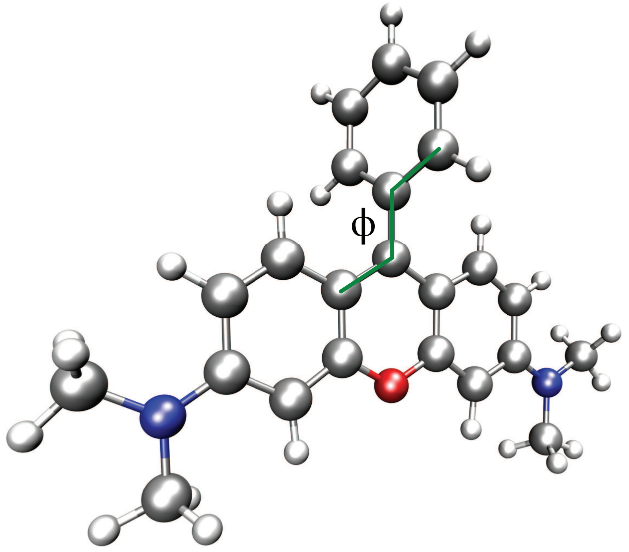

Figure S1: Fixed dihedral angle relaxed rotation profile. Chosen to be positive

Table S1: Optimized dihedral angles[a]

Name	ϕ
1-O	113.1
1-S	90.0
1-Se	90.0
1-Te	90.0
2-O	123.1
2-S	103.2
2-Se	90.0
2-Te	90.0

[a] ϕ in degrees, see Figure S1.

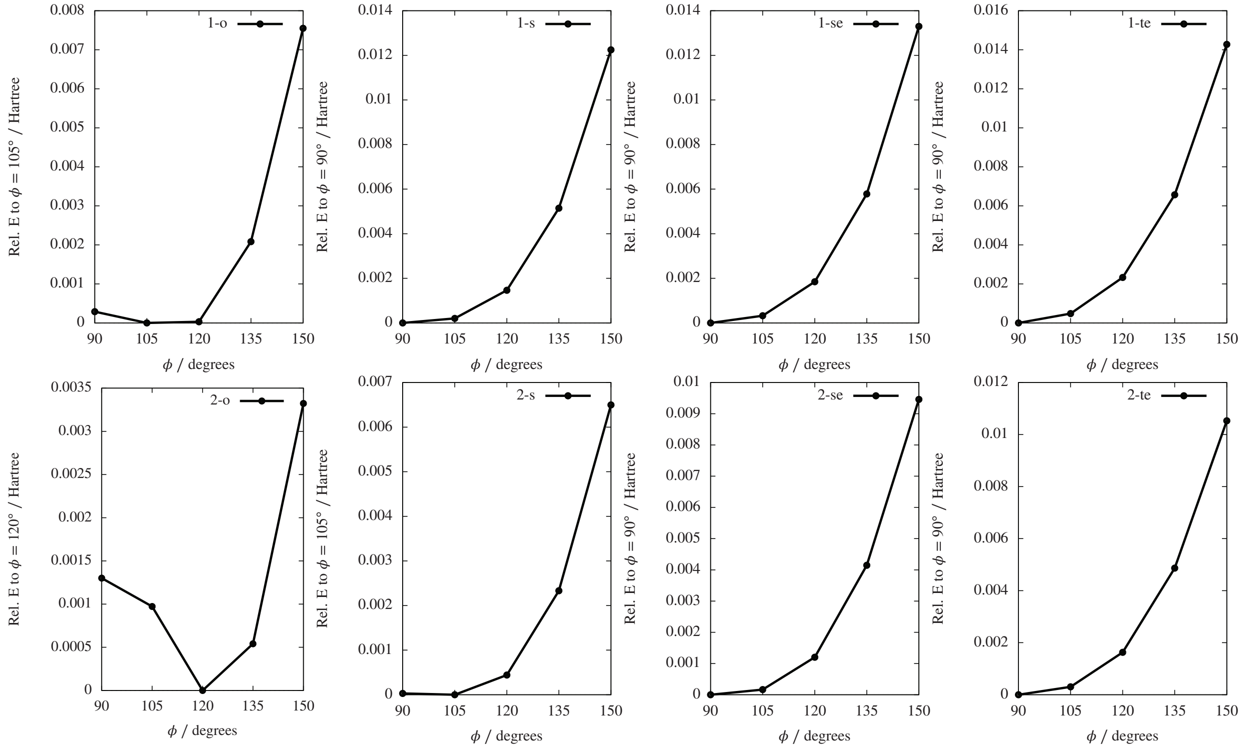

Figure S2: The relaxed rotation profile results.

2 Excitation Comparison between RRP and Symmetrized Geometries

To facilitate reasonable EOM-CCSD(T) energies, the optimized structures were symmetrized and truncated. In Table S2, the angle ϕ (see Figure S1) was set to 90° and optimized with symmetry (C_{2v} for benzene and C_s for thiophene structures). Additionally, the symmetrized structures were truncated by removing the benzene and thiophene moeities and -CH_3 groups from the nitrogens followed by optimization with C_{2v} symmetry.

Table S2: Excitation energies for symmetrized 1-O, 2-O, and 2-S.[a]

	PBE		LC-PBE	
	Opt.	Sym.	Opt.	Sym.
1-O	2.50	2.52	2.97	2.98
2-O	2.42	2.44	2.91	2.90
2-S	2.37	2.36	2.83	2.79

[a] Energies given in eV. "Opt." refers to the optimized and "Sym." refers to the symmetrized structures with $\phi = 90°$.

Table S3: Singlet excitations for 1,2-E versus truncated structures.[a]

	1-E		2-E		Truncated		Difference (1-E, 2-E)	
Name	PBE	LC*	PBE	LC*	PBE	LC*	PBE	LC*
O	2.52	2.80	2.44	2.72	2.86	3.14	0.34, 0.42	0.34, 0.42
S	2.44	2.70	2.36	2.65	2.77	3.05	0.33, 0.41	0.35, 0.40
Se	2.41	2.67	2.33	2.60	2.74	3.02	0.33, 0.41	0.35, 0.42
Te	2.37	2.62	2.30	2.55	2.70	2.99	0.33, 0.41	0.37, 0.44

[a] Energies in eV.

Table S4: Triplet excitations for 1,2-E versus truncated structures.[a]

	1-E		2-E		Truncated		Difference (1-E, 2-E)	
Name	PBE	LC*	PBE	LC*	PBE	LC*	PBE	LC*
O	1.63	1.75	1.55	1.66	1.82	1.88	0.19, 0.27	0.13, 0.22
S	1.60	1.71	1.52	1.62	1.81	1.87	0.22, 0.29	0.16, 0.24
Se	1.58	1.70	1.51	1.61	1.81	1.86	0.23, 0.30	0.17, 0.25
Te	1.56	1.66	1.49	1.57	1.79	1.83	0.24, 0.30	0.17, 0.26

[a] Energies in eV.

3 Tuning Data

γ is here the range separation parameter in an error-function separation of r_{12}.

Table S5: Optimally tuned γ values for relaxed geometries[a]

name	γ^*
1-O	0.182
1-S	0.179
1-Se	0.177
1-Te	0.175
2-O	0.182
2-S	0.180
2-Se	0.178
2-Te	0.175

[a] All values in au^{-1}

Table S6: Optimally tuned γ values for truncated geometries[a]

name	γ^*
O	0.224
S	0.219
Se	0.216
Te	0.225

[a] All values in au^{-1}

4 Additional Excitation Data and Plots

Table S7: Numerical analysis of integrals involved in TDDFT calculations of the HOMO-LUMO excitation of 1,2-E series. Coulomb and exchange integrals are computed from HOMO and LUMO orbitals with PBE and TZP basis set.[a]

Integral	1-O	1-S	1-Se	1-Te	2-O	2-S	2-Se	2-Te
$[pp\|r_{12}^{-1}\|qq]$	4.530	4.437	4.414	4.382	4.508	4.419	4.396	4.365
$[pq\|r_{12}^{-1}\|pq]$	1.053	1.026	1.025	1.036	1.077	1.050	1.049	1.059
$[pp\|r_{12}^{-1}\|pp]$	5.078	5.025	5.010	4.987	5.073	5.018	5.003	4.979
$[qq\|r_{12}^{-1}\|qq]$	5.612	5.457	5.398	5.293	5.544	5.385	5.325	5.219
$[pq\|f_{\mathrm{XC}}^{\alpha\alpha}\|pq]$	-0.116	-0.110	-0.109	-0.109	-0.117	-0.112	-0.111	-0.110
$[pp\|f_{\mathrm{XC}}^{\alpha\alpha}\|pp]$	-0.293	-0.293	-0.292	-0.290	-0.292	-0.292	-0.291	-0.289
$[qq\|f_{\mathrm{XC}}^{\alpha\alpha}\|qq]$	-0.340	-0.326	-0.321	-0.312	-0.327	-0.313	-0.307	-0.298
$[pq\|f_{\mathrm{XC}}^{\alpha\alpha} + f_{\mathrm{XC}}^{\alpha\beta}\|pq]$	-0.133	-0.126	-0.125	-0.125	-0.135	-0.128	-0.127	-0.126
$[pp\|f_{\mathrm{XC}}^{\alpha\alpha} + f_{\mathrm{XC}}^{\alpha\beta}\|pp]$	-0.334	-0.334	-0.333	-0.331	-0.333	-0.333	-0.332	-0.330
$[qq\|f_{\mathrm{XC}}^{\alpha\alpha} + f_{\mathrm{XC}}^{\alpha\beta}\|qq]$	-0.393	-0.377	-0.372	-0.361	-0.378	-0.361	-0.356	-0.345
$[pq\|f_{\mathrm{XC}}^{\alpha\alpha} - f_{\mathrm{XC}}^{\alpha\beta}\|pq]$	-0.099	-0.094	-0.093	-0.093	-0.100	-0.095	-0.094	-0.094
$[pp\|f_{\mathrm{XC}}^{\alpha\alpha} - f_{\mathrm{XC}}^{\alpha\beta}\|pp]$	-0.251	-0.251	-0.250	-0.249	-0.251	-0.250	-0.250	-0.248
$[qq\|f_{\mathrm{XC}}^{\alpha\alpha} - f_{\mathrm{XC}}^{\alpha\beta}\|qq]$	-0.288	-0.275	-0.271	-0.262	-0.277	-0.264	-0.259	-0.251
$\frac{1}{2}[\Delta\rho\|r_{12}^{-1} + f_{\mathrm{XC}}^{\alpha\alpha}\|\Delta\rho]$	0.615	0.605	0.593	0.565	0.608	0.592	0.580	0.551
$\frac{1}{2}[\Delta\rho\|f_{\mathrm{XC}}^{\alpha\alpha}\|\Delta\rho]$	-0.201	-0.199	-0.197	-0.192	-0.192	-0.191	-0.188	-0.183
$\langle\|\varphi_p\|\|\|\varphi_q\|\rangle$	0.667	0.653	0.652	0.654	0.674	0.663	0.661	0.663

[a] All values in eV, except $\langle|\varphi_p|||\varphi_q|\rangle$ which is unitless.

References

[1] Pepe, G.; Cole, J. M.; Waddell, P. G.; Perry, J. I. *Mol. Syst. Des. Eng.* **2016,** *1,* 416–435.

Table S8: Excitation energies and S/T gap for truncated dyes in a 2 level model[a]

	$^1\Delta E$		$^3\Delta E$		S/T Gap	
	PBE	LC-PBE*	PBE	LC-PBE*	PBE	LC-PBE*
O	4.21	4.11	1.86	2.23	2.35	1.89
S	4.14	4.05	1.85	2.20	2.29	1.85
Se	4.11	4.02	1.84	2.19	2.27	1.84
Te	4.09	4.00	1.83	2.18	2.27	1.83

[a] All values in eV.

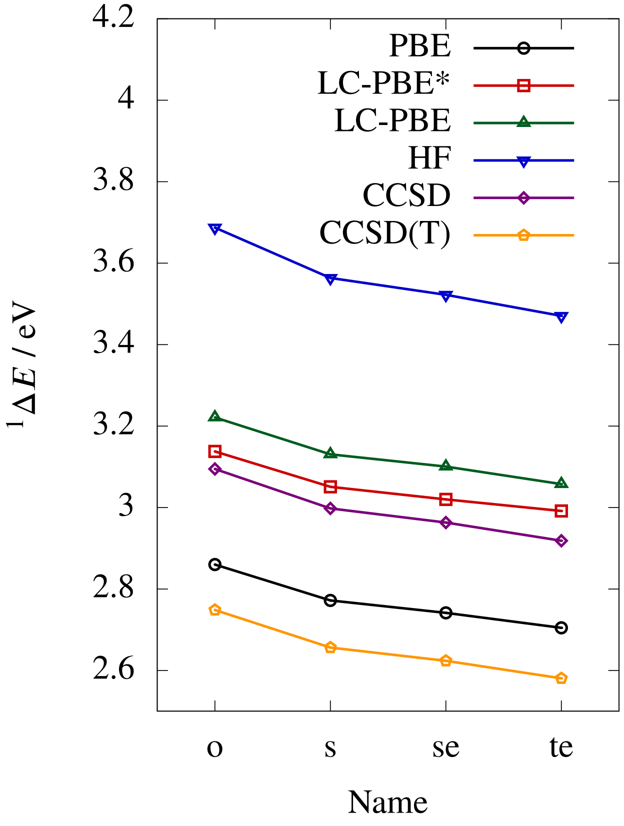

Figure S3: Singet excitation energies at various levels of theory.

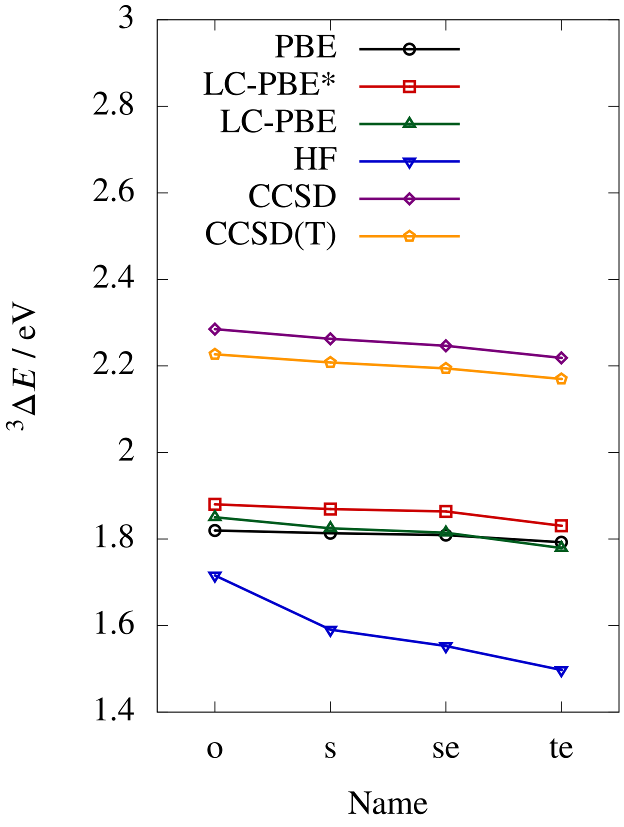

Figure S4: Triplet excitation energies at various levels of theory.

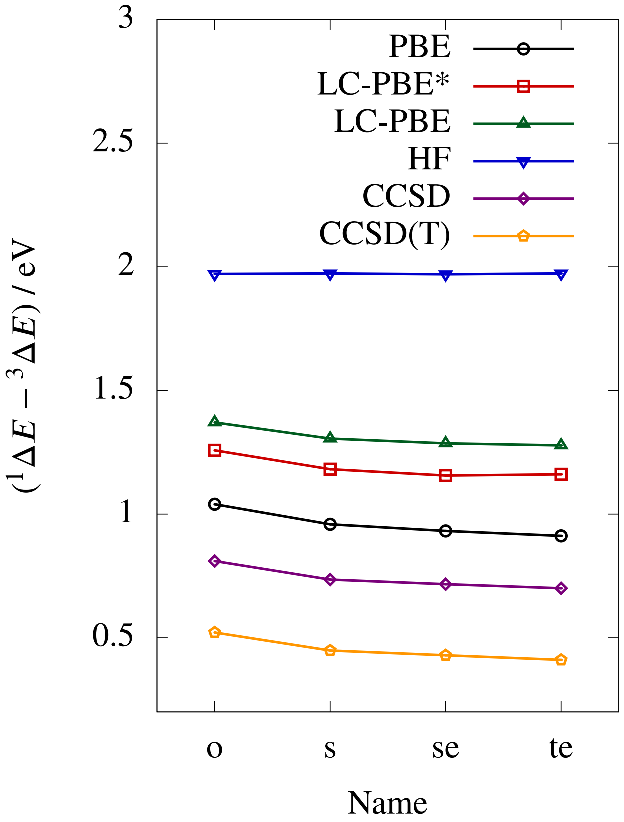

Figure S5: S/T gaps for truncated dyes from Table 2.

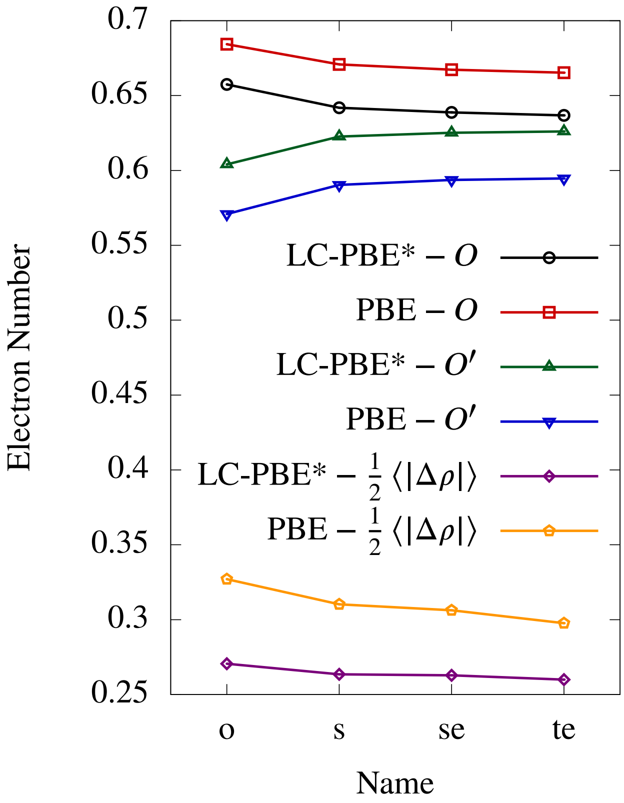

Figure S6: CT criterion from Table 3

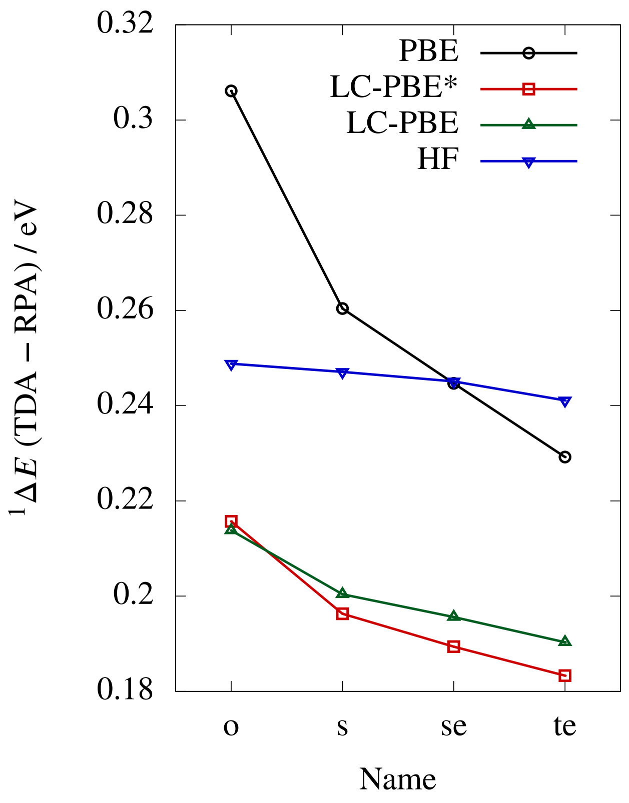

Figure S7: TDA − full TDKST differences for $^1\Delta E$ from Table 6.

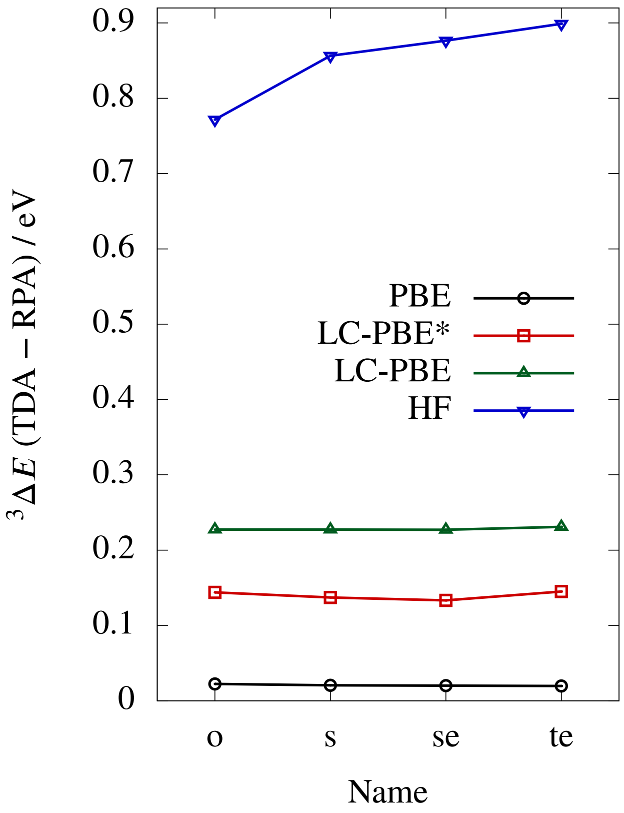

Figure S8: $^3\Delta E$ TDA − full TDKST differences from Table 6.
